# Multiple Approaches to Improve the Quality of Cereal-Based Foods

**DOI:** 10.3390/foods11131849

**Published:** 2022-06-23

**Authors:** Barbara Laddomada, Weiqun Wang

**Affiliations:** 1 Institute of Sciences of Food Production (ISPA), National Research Council (CNR), Via Monteroni, 73100 Lecce, Italy; 2 Department of Food, Nutrition, Dietetics and Health, Kansas State University, Manhattan, KS 66506, USA; wwang@ksu.edu

The interest in improving the health benefits of cereal foods is continuously increasing. This is essentially due to the high frequency of their consumption worldwide, and to the chance of using them to vehicle health promoting components in the diet that may counteract the occurrence of non-communicable diseases (NCDs). This would contribute to improving health maintenance and disease prevention on line with aims of the 2030 Agenda for Sustainable Development that recognizes NCDs as a “major challenge for sustainable development”. Incorrect dietary behaviors are among the major “behavioral risk factors” in NCDs incidences. In fact, the increasing consumption of ready meals that are rich in sugars and lipids, and poor in bioactive compounds and fibers, is contributing to overweight and obesity worldwide, with an increasing trend in low-, middle-, and even high-income countries.

Improving the functional and technological properties of cereal wholemeal flours is a first and direct goal in favor of the production and spread of cereal-based foods with improved functional skills. The challenge is to improve the cereal foods not only for health, but also for taste in order to guarantee consumer’s acceptance, and the economic potential of the new products. This aim can be achieved using different strategies, some being based on the selection of the best genetic materials and on breeding programs, some others focusing on the upgrade of milling processes or on using new foods formulations. 

The Special Issue “Functional Cereal Foods for Health Benefits: Genetic and/or Processing Strategies to Enhance the Quali-Quantitative Composition of Bioactive Components” collects 12 original research articles on different innovative approaches to improve the health potential of cereal foods contributing to a Mediterranean diet-based lifestyle. Some of the research articles address the issue of finding the best milling strategies to produce wholemeal flour with improved contents of bioactive components, but also reduced contents of contaminants and toxic components. Above all, the aim is to improve the health value of the end-products while maintaining good technological and sensorial quality. The latter issues seemed to be not completely overcome when different pilot-scale milling methods were tested to produce whole-wheat flour to be used for Chinese Steamed Bread (CSB) and Chinese leavened pancakes (CLP) [1]. In fact, dietary fibers increased by 1.6 fold, and ferulic acid by 1.9 fold, but such improvements were accompanied by an increase of damaged starch percentage, and overall affected the quality of end-products [1]. The development of an upgraded micronization plant and of a modified air classification plant able to produce different types of durum wheat milling fractions enriched in bran particles and consequently of phenolic bioactive compounds was also presented in this special issue [2]. This innovative solution offered the advantage of directly producing different unrefined milling products with peculiar features, making them suitable to produce diverse types of unrefined end-products (pasta, bread, biscuits, etc.). Due to the presence of bran particles in different rates within each air-classified fraction, the millings had a higher technological quality compared to that of semolina supplemented with the addition of bran aliquots. Nevertheless, the alvegraphic behavior of the unrefined millings displays a significant reduction in the alveographic parameters, especially with regard to a P/L increase [3]. This technology has the advantage of reducing the levels of organic and inorganic contaminants, thus reducing health hazards to consumers [4].

Another aspect of the special issue is about the effects of seed fermentation or dehulling on improving the concentration of nutritional and bioactive compounds. As an example, xylanase and cellulose, produced by *Aspergillus awamori* and *Thricoderma reesei*, were used on rice seeds [5]. In the selected conditions, such treatment facilitated a consistent increase of Ca, P, Iron, free amino acids, phenolic compounds and proteins. Another application of fermentation regarded coix seeds by using *Monascus purpureus* [6]. As a result, the contents of tocols, γ-oryzanol, and coixenolide increased improving also the associated antioxidant and anticancer activity on laryngeal carcinomatous HEp2 cells [6]. 

Dehulling also could have an indirect effect on the improvement of phenolic compounds with health benefits [7]. In fact, the germination of dehulled buckwheat seeds resulted in 1.8-fold and 1.9-fold higher phenolics and antioxidant activity compared to hulled seeds [7].

Ultimately, as a third topic, this special issue includes studies investigating the effect of extreme environmental conditions on the accumulation of phenolic acids in the wholemeal flour of different durum wheat cultivars [8]. Good yield performances and high accumulation of bioactive compounds across highly differing growing conditions could be significant to improve both the durum wheat resilience and health-promoting value. This in agreement with Goal 13 of the 2030 Agenda requiring urgent actions to combat climate change and its impacts. 

Notably, cereal polyphenols can be found associated with cancer prevention, though more studies are needed in order to receive specific health claims by FDA and EFSA. To this aim, phenolic extracts from sorghum with black pericarp were tested on the inhibition of hepatocarcinoma HepG2 cells and colorectal adenocarcinoma Caco-2 cells [9]. The cell growth inhibition by the sorghum phenolic extracts was significantly associated with their phenolic content and the inhibition appeared to be mediated by cytostatic and apoptotic mechanisms rather than cytotoxicity [9].

The quality of the majority of cereal foods depends largely on protein composition. In wheat, especially the high and low molecular weight glutenins have a great role on that. The effect of these proteins was analyzed gene-by-gene or as haplotypes providing new insights in the importance of common and rare allelic variants to improve gluten quality [10]. As a matter of fact, the biological value of cereal proteins is limited by the deficiency of essential amino acids. To counteract malnutrition due to a poorly balanced diet, it is urgent to select cereals that, besides having high yield potential, contain higher amounts of essential amino acids. Such a work was carried out in maize and is particularly valuable in view of future hybridization to produce highly nutritious maize hybrids to address malnutrition caused by a maize-based diet [11].

Finally, this special issue includes three reviews discussing the nutritional significance of wheat proteins on human health [12] and of their evolution from wheat domestication to modern cultivars [13]. The reviews particularly address common misconceptions that are associated with wheat consumption in relation to health; the latter provides a holistic view of the temporal and proteogenomic evolution of wheat from its domestication to the massively produced high-yield crop of our day [12,13].

In relation to the celiac disease and gluten sensitivity, sorghum grains can enter gluten-free diets being also interesting for their potential phenolic-induced health benefits [14]. The third review covers aspects of sorghum health benefits and explores their mechanisms of action [14].

In conclusion, this Special Issue offers an interesting contribution to the field providing readers information on multiple approaches to improve the cereal-based foods. The research articles and the reviews can be useful both for researchers and for industry operators. In most cases, these strategies may alter the technological and sensory properties, but confer different quality characteristics that, when explained, can be accepted by consumers and producers.
